# *H*_*∞*_ Time-Delayed Fractional Order Adaptive Sliding Mode Control for Two-Wheel Self-Balancing Vehicles

**DOI:** 10.1155/2020/4529131

**Published:** 2020-08-10

**Authors:** Han Xue, Qionglin Fang, Jifeng Zhong, Zhe-ping Shao

**Affiliations:** ^1^School of Navigation, Jimei University, Xiamen 361021, Fujian, China; ^2^National and Local Joint Engineering Research Center of Ship Aided Navigation Technology, Xiamen 361021, Fujian, China; ^3^Fujian Shipping Research Institute, Xiamen 361021, Fujian, China; ^4^Xiamen Southeast International Shipping Research Center, Xiamen 361021, Fujian, China

## Abstract

In this paper, a time-delayed fractional order adaptive sliding mode control algorithm is proposed for a two-wheel self-balancing vehicle system. The closed-loop system is proved based on the Lyapunov-Razumikhin function. The switching function is designed to make the system robust when facing uncertainties and external disturbances. It is designed to avoid monotonically increasing gains and can handle state-dependent uncertainties without a prior bound. The two-wheel self-balancing vehicle used in the experiment consists of a gyroscope MPU-6050 and accelerometer, a motor driving circuit composed of a motor driving chip TB6612FNG, and STM32F103x8B that is selected as the control core. The experimental results show that the time-delayed fractional order adaptive sliding mode control algorithm can make the vehicle achieve autonomous balance and quickly restore its stable state while appropriate disturbance is introduced.

## 1. Introduction

The movement principle of a two-wheel self-balancing vehicle is based mainly on the basic principle of dynamic stability. The gyroscope and acceleration sensor inside the vehicle are used to detect the changing attitude of the vehicle. The control system is used to precisely adjust the motor to maintain the balance of the system. At present, two-wheel self-balancing vehicles are widely used in transportation, rescue, and other fields. They have provided an effective solution to environmental pollution and energy crisis. Two-wheel self-balancing vehicles have the characteristics of high order, nonlinearity, strong coupling, and underactuation. However, some uncertainties, such as mechanism friction, ground friction, changing payload, and road gradient still need to be dealt with. At present, the control algorithms used in two-wheel self-balancing vehicles include PID, neural network, mode predictive control, *O* control, and so on.

Sliding mode control is a branch of variable structure control that was proposed in the 1950s. It belongs to a nonlinear control that is realized through a switching function. It is insensitive to parameter changes and has the capability of rejecting interference. There are many methods to deal with the considered external disturbance. Li investigated the problem of induced L2 disturbance attenuation control design for T-S fuzzy delta operator systems with time-varying delays via the IO approach [1]. The time delay was handled by the IO approach to facilitate the establishment of the feedback interconnection system. Based on the feedback interconnection system, a sufficient condition of asymptotical stability was obtained for the closed-loop system. The desired DOF controller was designed to guarantee the closed-loop system to be asymptotically stable with an induced L2 disturbance attenuation performance. Liu studied the finite-time H∞ control of a fractional order HTGS [2]. Based on the generalized T-S fuzzy model, the fractional order fuzzy model of a HTGS was presented. By combining finite-time control and H∞ control theory, a finite-time H∞ state feedback control was proposed for the HTGS. The control is based on the fractional order stability theorem. Gao presented a novel descriptor SMO to formulate accurate estimations of both plant states and actuator fault deviation [3]. The stability of error dynamics was analyzed and a developed stability criterion was established. The developed criterion gave a solvable solution to obtain the observer gains using convex optimization algorithm. A linear sliding surface and integral sliding surface were designed. A new SMC law was formulated with a discontinuous control input and an equivalent control to guarantee that the finite-time convergence of the plant trajectories. Liu proposed an ESO-based cascade controller for regulating the oxygen excess ratio of the PEMFC air-feed system to its desired value, using the sliding mode technique [4]. The control objective was to avoid oxygen starvation during sudden load changes. The designed cascade controller consists of oxygen excess ratio tracking outer loop and compressor flow rate regulation inner loop. The ESO was used to reconstruct the oxygen excess ratio. The outer control loop using the estimated oxygen excess ratio provided the compressor flow rate reference for the inner loop based on the STA. A simple SMC law consisting of a linear term and a switching term was designed for the inner loop.

In recent years, sliding mode control has developed rapidly in the field of two-wheel self-balancing robots. Abeygunawardhana proposed a second-order sliding mode controller based on the disturbance observer [5]. Rafael established a reference model based on a dual-relay controller for tracking control problems of a wheel pendulum. [6] Guo proposed a sliding mode controller for an underactuated system consisting of a pendulum and two wheels. [7] Based on the nonlinear dynamic model, Yue divided the whole system into three subsystems: rotational motion, longitudinal motion, and zero dynamics. [8] Within these subsystems, the inclined angle of the vehicle was treated as zero dynamics, the longitudinal acceleration was used as control input, and the sliding mode control technology was used to stabilize the zero dynamic subsystem. Li established a physical model of a two-wheel self-balancing vehicle and designed a sliding mode controller based on Newtonian mechanics. [9] Dai used the sliding mode control to achieve self-balance and pitch angle control of a double-wheel inverted pendulum robot with friction compensation. [10] Ali used a set of highly coupled nonlinear differential equations to represent the motion model of two-wheel self-balancing robots [11]. Zhou proposed a robust integral sliding mode controller based on bounded system uncertainties with linearization error and input delay [12]. Nasim used back-stepping sliding mode to solve the problem of balancing and trajectory tracking of a two-wheel balanced mobile robot [13]. Chen proposed a robust tracking control based on a nonlinear disturbance observer for self-balanced mobile robots with unknown external disturbances [14]. Wang proposed a model-free fractional order sliding mode control based on an extended state observer, providing a framework that considered nonlinearity of friction, parameter variation, and external disturbance [15].

Designing robust controllers for uncertain time-delay systems has become a key problem. The robustness of sliding mode variable structure control makes it insensitive to the model error, changing parameters, and external disturbance. Therefore, variable structure control has become an effective control method for time-delay systems. With the development of variable structure control theory, some research is available on variable structure control of time-delay systems [16, 17]. Roy solved the long-standing problem of consistent stability analysis and control design in continuous time for time-delay control (TDC). Based on its newly proposed structure of TDE error, a more robust control law was formulated [18]. Roy made a number of significant contributions: proposing a novel adaptive sliding mode control (ASMC) methodology that does not require a priori bounded uncertainty [19], a novel ASMC strategy that overcomes underestimation and overestimation problems commonly observed in conventional ASMC [20], and a hybrid control methodology called a time-delay sliding mode control for accurate path tracking of nonholonomic wheeled mobile robots [21].

In this paper, an adaptive time-delay fractional order sliding mode control algorithm is proposed to control the motion of a two-wheel self-balancing vehicle. The stability of the closed-loop system is proved based on the quadratic Lyapunov function. The influence of control parameters on control performance is analyzed based on a simulation. The physical experiment of the two-wheel self-balancing vehicle is carried out to verify the effectiveness of the control algorithm. The technique contribution of the presented control method is summarized as follows:

1. A time-delay control (TDC) algorithm is proposed. The TDC approximates the system uncertainty by using control input and state information of the immediate past time instant.
2. A fractional order sliding mode control is designed. Fractional order sliding mode has a memory effect and better stability. Its parameter selection range is wider and more flexible. Fractional calculus operator can obtain faster speed and higher control accuracy. It not only makes the system converge in finite time but also effectively weakens the chattering of traditional integer order sliding mode controller.
3. Adaptive switching gain of the sliding mode control is designed.

## 2. Mathematical Model of TWIP

**Figure 1:**
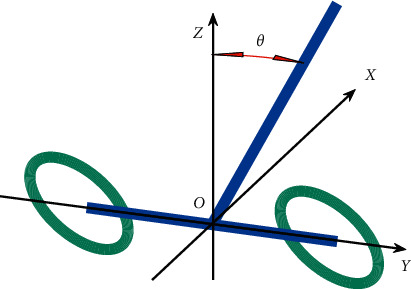
Model of a two-wheel self-balancing vehicle.

The coordinate system is established as follows: the midpoint of the line segment connecting the centroids of two wheels is set as the origin *X*. The moving direction of the vehicle is set as the *Y* axis. The line connecting the centroids of the two wheels is set as the axis. The vertical upward direction through the origin is set as the *Z* axis. The motion model of the two-wheel self-balancing vehicle is shown in Figure 1.

If we denote *M* as the mass of vehicle, *m* as the mass of wheel, *R* as the radius of wheels, *l* as the distance from the centroid of the vehicle to the origin, *J* as the moment of inertia of the vehicle, *J*_*w*_ as the moment of inertia of wheel, *θ* as the angle of inclination of the vehicle, and *x*_*m*_ as the displacement of vehicle, then the kinetic energy *K* of the vehicle is expressed as follows:

(1) $$\begin{array}{c} K=\frac{1}{2}M\left[{\left({\overset{˙}{x}}_{m}+l\overset{˙}{\theta }\cos\;\theta \right)}^{2}+{\left(l\overset{˙}{\theta }\sin\;\theta \right)}^{2}\right]+\frac{1}{2}J{\overset{˙}{\theta }}^{2}. \end{array}$$

The kinetic energy of wheels *K*_*w*_ is

(2) $$\begin{array}{c} K_{w}=\frac{1}{2}m{\overset{˙}{x}}_{m}^{2}+\frac{1}{2}J_{w}{\left(\frac{{\overset{˙}{x}}_{m}}{R}\right)}^{2}. \end{array}$$

The potential energy of system is given as follows:

(3) $$\begin{array}{c} P=Mgl\;\cos\;\theta . \end{array}$$

The Lagrange function is constructed as follows:

(4) $$\begin{array}{c} L=K+K_{w}-P\\ =\frac{1}{2}M\left[{\left({\overset{˙}{x}}_{m}+l\overset{˙}{\theta }\cos\;\theta \right)}^{2}+{\left(l\overset{˙}{\theta }\sin\;\theta \right)}^{2}\right]+\frac{1}{2}J{\overset{˙}{\theta }}^{2}+\frac{1}{2}m{\overset{˙}{x}}_{m}^{2}+\frac{1}{2}J_{w}{\left(\frac{{\overset{˙}{x}}_{m}}{R}\right)}^{2}-Mgl\;\cos\;\theta . \end{array}$$

Defining *u* as the control input of the system, we obtain the following using the Euler-Lagrange formula:

(5) $$\begin{array}{c} \frac{\text{d}}{\text{d}t}\left(\frac{\partial L}{\partial \overset{˙}{\theta }}\right)-\frac{\partial L}{\partial \theta }=-u, \end{array}$$

(6) $$\begin{array}{c} \frac{\text{d}}{\text{d}t}\left(\frac{\partial L}{\partial {\overset{˙}{x}}_{m}}\right)-\frac{\partial L}{\partial x_{m}}=\frac{u}{R}. \end{array}$$

Substituting (4) into (5), we obtain

(7) $$\begin{array}{c} \frac{\text{d}}{\text{d}t}\left(\frac{\partial L}{\partial \overset{˙}{\theta }}\right)-\frac{\partial L}{\partial \theta }\\ =\frac{\text{d}}{\text{d}t}\left[M\left({\overset{˙}{x}}_{m}+l\overset{˙}{\theta }\cos\;\theta \right)l\;\cos\;\theta +J\overset{˙}{\theta }+Ml^{2}\overset{˙}{\theta }\sin^{2}\;\theta \right]\\ +M\left({\overset{˙}{x}}_{m}+l\overset{˙}{\theta }\cos\;\theta \right)l\overset{˙}{\theta }\sin\;\theta -Ml^{2}{\overset{˙}{\theta }}^{2}\sin\;\theta \;\cos\;\theta -Mgl\;\sin\;\theta \\ =M{\overset{¨}{x}}_{m}l\;\cos\;\theta -M{\overset{˙}{x}}_{m}l\;\sin\;\theta \overset{˙}{\theta }-2Ml^{2}\sin\;\theta \;\cos\;\theta {\overset{˙}{\theta }}^{2}\\ +Ml^{2}\overset{¨}{\theta }\cos^{2}\;\theta +J\overset{¨}{\theta }+Ml^{2}\overset{¨}{\theta }\sin^{2}\;\theta +2Ml^{2}{\overset{˙}{\theta }}^{2}\sin\;\theta \;\cos\;\theta \\ +M\left({\overset{˙}{x}}_{m}+l\overset{˙}{\theta }\cos\;\theta \right)l\overset{˙}{\theta }\sin\;\theta -Ml^{2}{\overset{˙}{\theta }}^{2}\sin\;\theta \;\cos\;\theta -Mgl\;\sin\;\theta \\ =M{\overset{¨}{x}}_{m}l\;\cos\;\theta +J\overset{¨}{\theta }+Ml^{2}\overset{¨}{\theta }-Mgl\;\sin\;\theta =-u. \end{array}$$

Substituting (4) into (6), we obtain

(8) $$\begin{array}{c} \frac{\text{d}}{\text{d}t}\left(\frac{\partial L}{\partial {\overset{˙}{x}}_{m}}\right)-\frac{\partial L}{\partial x_{m}}\\ =\frac{\text{d}}{\text{d}t}\left[M\left({\overset{˙}{x}}_{m}+l\overset{˙}{\theta }\cos\;\theta \right)+m{\overset{˙}{x}}_{m}+J_{w}\frac{{\overset{˙}{x}}_{m}}{R^{2}}\right]\\ =\left(M+m+\frac{J_{w}}{R^{2}}\right){\overset{¨}{x}}_{m}+Ml\overset{¨}{\theta }\cos\;\theta -Ml{\overset{˙}{\theta }}^{2}\sin\;\theta =\frac{u}{R}. \end{array}$$

By simplifying and linearizing (6) and (7), we obtain

(9) $$\begin{array}{c} \overset{¨}{\theta }=\frac{Mlg\left(J_{w}+mR^{2}+MR^{2}\right)\theta }{JJ_{w}+R^{2}mJ+JR^{2}M+Ml^{2}J_{w}+R^{2}Ml^{2}m}-\frac{J_{w}+mR^{2}+MR^{2}+RMl}{JJ_{w}+R^{2}mJ+JR^{2}M+Ml^{2}J_{w}+R^{2}Ml^{2}m}u, \end{array}$$

(10) $$\begin{array}{c} {\overset{¨}{x}}_{m}=\frac{-M^{2}l^{2}R^{2}g\theta +\left(J_{w}R+MlR^{2}+Ml^{2}R\right)u}{JJ_{w}+R^{2}mJ+JR^{2}M+Ml^{2}J_{w}+R^{2}Ml^{2}m}. \end{array}$$

The state variables are given as follows:

(11) $$\begin{array}{c} x={\left[\theta ,x_{m}\right]}^{T}. \end{array}$$

Define

(12) $$\begin{array}{c} f=\left[\frac{Mlg\left(J_{w}+mR^{2}+MR^{2}\right)\theta }{JJ_{w}+R^{2}mJ+JR^{2}M+Ml^{2}J_{w}+R^{2}Ml^{2}m}\frac{-M^{2}l^{2}R^{2}g\theta }{JJ_{w}+R^{2}mJ+JR^{2}M+Ml^{2}J_{w}+R^{2}Ml^{2}m}\right]. \end{array}$$

Define the input matrix as follows:

(13) $$\begin{array}{c} B=\left[-\frac{J_{w}+mR^{2}+MR^{2}+RMl}{JJ_{w}+R^{2}mJ+JR^{2}M+Ml^{2}J_{w}+R^{2}Ml^{2}m}\frac{J_{w}R+MlR^{2}+Ml^{2}R}{JJ_{w}+R^{2}mJ+JR^{2}M+Ml^{2}J_{w}+R^{2}Ml^{2}m}\right]. \end{array}$$

Thus, the state space can be written as follows:

(14) $$\begin{array}{c} \overset{¨}{x}\left(t\right)=f\left(t\right)+Bu\left(t\right). \end{array}$$

Considering the existence of external disturbance *d*, the system dynamics can be written as follows:

(15) $$\begin{array}{c} \overset{¨}{x}\left(t\right)=f\left(t\right)+d\left(t\right)+Bu\left(t\right). \end{array}$$

If we define

(16) $$\begin{array}{c} \xi \left(t\right)=f\left(t\right)+d\left(t\right), \end{array}$$

(17) $$\begin{array}{c} \tau \left(t\right)=Bu\left(t\right), \end{array}$$

then (15) can be written as follows:

(18) $$\begin{array}{c} \overset{¨}{x}\left(t\right)=\xi \left(t\right)+\tau \left(t\right). \end{array}$$

## 3. Time-Delayed Fractional Order Adaptive Sliding Mode Control

### 3.1. Fractional Order Calculus

Fractional calculus is the operation of derivatives and integrals extended to the fractional order. It actually provides a more precise tool for describing physical systems. The Riemann-Liouville (RL) fractional differintegral is one of its most common definitions. For a function *x* that is defined in [*t*_0_, *t*], the RL fractional integrator is defined as follows [22]:

(19) Dt0tαxt=1Γα∫t0tt−τα−1xτdτ,

where *α* is the fractional order, *τ* is the integral variable, and Γ(*x*) is the gamma function that is defined as follows:

(20) $$\begin{array}{c} \Gamma \left(x\right)=\int_{0}^{\infty }e^{-t}t^{x-1}\text{d}t. \end{array}$$

The RL fractional derivative is defined as follows:

(21) Dt0tαxt=dmdtn1Γm−α∫t0tt−τm−α−1xτdτ,

where *α* ∈ [*m* − 1, *m*) and *m* is a positive integer near *α*.

### 3.2. The Proposed Algorithm

Let *x*_*d*_ be the desired state and $\tilde{x}$ be the state error:

(22) $$\begin{array}{c} \tilde{x}\left(t\right)=x_{d}\left(t\right)-x\left(t\right). \end{array}$$

Then, the following sliding mode surface function can be constructed:

(23) $$\begin{array}{c} s\left(t\right)=\overset{˙}{\tilde{x}}\left(t\right)+cD^{\alpha }\tilde{x}\left(t\right), \end{array}$$

where 0 < *α* < 1, *c* > 0.

Taking the derivative of formula (23) yields

(24) $$\begin{array}{c} \overset{˙}{s}\left(t\right)=\overset{¨}{\tilde{x}}\left(t\right)+cD^{\alpha +1}\tilde{x}\left(t\right). \end{array}$$

Substituting (22) into (24), one can obtain

(25) $$\begin{array}{c} \overset{˙}{s}\left(t\right)={\overset{¨}{x}}_{d}\left(t\right)-\overset{¨}{x}\left(t\right)+cD^{\alpha +1}\tilde{x}\left(t\right). \end{array}$$

Substituting (21) into (25), one can obtain

(26) $$\begin{array}{c} \overset{˙}{s}\left(t\right)={\overset{¨}{x}}_{d}\left(t\right)-\xi \left(t\right)-\tau \left(t\right)+cD^{\alpha +1}\tilde{x}\left(t\right). \end{array}$$

Denote *h* as the time delay. The TDC approximates the system uncertainty by using control input and state information of the immediate past time instant. Then, (18) can be written as follows:

(27) $$\begin{array}{c} \xi \left(t-h\right)=\overset{¨}{x}\left(t-h\right)-\tau \left(t-h\right). \end{array}$$

The adaptive control law is constructed as follows:

(28) $$\begin{array}{c} \tau \left(t\right)={\overset{¨}{x}}_{d}\left(t\right)-\xi \left(t-h\right)+cD^{\alpha +1}\tilde{x}\left(t\right)+\rho \left(t\right)\frac{s\left(t\right)}{\left\|s\left(t\right)\right\|}. \end{array}$$

The switching gain satisfies

(29) $$\begin{array}{c} \rho \left(t\right)={\hat{K}}_{0}\left(t\right)+{\hat{K}}_{1}\left(t\right)\left\|x\left(t\right)\right\|, \end{array}$$

(30) $$\begin{array}{c} {\overset{˙}{\hat{K}}}_{0}\left(t\right)=\left\|s\left(t\right)\right\|-\alpha_{0}{\hat{K}}_{0}\left(t\right), \end{array}$$

(31) $$\begin{array}{c} {\overset{˙}{\hat{K}}}_{1}\left(t\right)=\left\|s\left(t\right)\right\|\cdot \left\|x\left(t\right)\right\|-\alpha_{1}{\hat{K}}_{1}\left(t\right), \end{array}$$

where $\alpha_{0}>0,\alpha_{1}>0,{\hat{K}}_{0}\left(t\right)>0,{\hat{K}}_{1}\left(t\right)>0$.

### 3.3. Stability Analysis

**Theorem 1 .**

Based on the Lyapunov's stability theory, considering the kinematics equation (14) of a two-wheel self-balancing vehicle system with input delay and the adaptive sliding mode controller (28)–(31), the tracking error of the system converges and the system becomes uniform ultimate bounded.

**Proof**

Define

(32) $$\begin{array}{c} E=\left[\tilde{x}\overset{˙}{\tilde{x}}\right]. \end{array}$$

Construct a Lyapunov-Krasovsky functional as follows:

(33) $$\begin{array}{c} V\left(E,t\right)=\frac{1}{2}s^{T}\left(t\right)s\left(t\right)+\frac{1}{2}\overset{1}{\underset{i=0}{\sum }}{\left[{\hat{K}}_{i}\left(t\right)-K_{i}^{*}\right]}^{2}, \end{array}$$

where *K*_*i*_^*∗*^(*t*) > 0.

Taking the derivative of formula (33), one can obtain

(34) $$\begin{array}{c} \overset{˙}{V}\left(E,t\right)={\overset{˙}{s}}^{T}\left(t\right)s\left(t\right)+\overset{1}{\underset{i=0}{\sum }}\left[{\hat{K}}_{i}\left(t\right)-K_{i}^{*}\right]{\overset{˙}{\hat{K}}}_{i}\left(t\right). \end{array}$$

Substituting (26) into (34), one can obtain

(35) $$\begin{array}{c} \overset{˙}{V}\left(E,t\right)={\left[{\overset{¨}{x}}_{d}\left(t\right)-\xi \left(t\right)-\tau \left(t\right)+cD^{\alpha +1}\tilde{x}\left(t\right)\right]}^{T}s\left(t\right)+\overset{1}{\underset{i=0}{\sum }}\left[{\hat{K}}_{i}\left(t\right)-K_{i}^{*}\right]{\overset{˙}{\hat{K}}}_{i}\left(t\right). \end{array}$$

Substituting (28) into (35), one can obtain

(36) $$\begin{array}{c} \overset{˙}{V}\left(E,t\right)={\left[{\overset{¨}{x}}_{d}\left(t\right)-\xi \left(t\right)-{\overset{¨}{x}}_{d}\left(t\right)+\xi \left(t-h\right)-cD^{\alpha +1}\tilde{x}\left(t\right)-\rho \left(t\right)\frac{s\left(t\right)}{\left\|s\left(t\right)\right\|}+cD^{\alpha +1}\tilde{x}\left(t\right)\right]}^{T}s\left(t\right)+\overset{1}{\underset{i=0}{\sum }}\left[{\hat{K}}_{i}\left(t\right)-K_{i}^{*}\right]{\overset{˙}{\hat{K}}}_{i}\left(t\right)\\ ={\left[-\xi \left(t\right)+\xi \left(t-h\right)-\rho \left(t\right)\frac{s\left(t\right)}{\left\|s\left(t\right)\right\|}\right]}^{T}s\left(t\right)+\overset{1}{\underset{i=0}{\sum }}\left[{\hat{K}}_{i}\left(t\right)-K_{i}^{*}\right]{\overset{˙}{\hat{K}}}_{i}\left(t\right)\\ =-\left[\xi \left(t\right)-\xi {\left(t-h\right)}^{T}\right]s\left(t\right)-\rho \left(t\right)\left\|s\left(t\right)\right\|\\ +\overset{1}{\underset{i=0}{\sum }}\left[{\hat{K}}_{i}\left(t\right)-K_{i}^{*}\right]{\overset{˙}{\hat{K}}}_{i}\left(t\right). \end{array}$$

The approximation error is assumed to be bounded by

(37) $$\begin{array}{c} \left\|\xi \left(t\right)-\xi \left(t-h\right)\right\|\leq \delta \left\|s\left(t\right)\right\|, \end{array}$$

where *δ* > 0.

Substituting (29) into (34), one can obtain

(38) $$\begin{array}{c} \begin{array}{c} \overset{˙}{V}\left(E,t\right)=-{\left[\xi \left(t\right)-\xi \left(t-h\right)\right]}^{T}s\left(t\right)-\left[{\hat{K}}_{0}\left(t\right)+{\hat{K}}_{1}\left(t\right)\left\|x\left(t\right)\right\|\right]\left\|s\left(t\right)\right\|+\overset{1}{\underset{i=0}{\sum }}\left[{\hat{K}}_{i}\left(t\right)-K_{i}^{*}\right]{\overset{˙}{\hat{K}}}_{i}\left(t\right)\\ =-{\left[\xi \left(t\right)-\xi \left(t-h\right)\right]}^{T}s\left(t\right)-\overset{1}{\underset{i=0}{\sum }}{\hat{K}}_{i}\left(t\right){\left\|x\left(t\right)\right\|}^{i}\left\|s\left(t\right)\right\|+\overset{1}{\underset{i=0}{\sum }}\left[{\hat{K}}_{i}\left(t\right)-K_{i}^{*}\right]{\overset{˙}{\hat{K}}}_{i}\left\|s\left(t\right)\right\|\\ \leq -{\left[\xi \left(t\right)-\xi \left(t-h\right)\right]}^{T}s\left(t\right)-\overset{1}{\underset{i=0}{\sum }}\left[{\hat{K}}_{i}\left(t\right)-K_{i}^{*}\right]{\left\|x\left(t\right)\right\|}^{i}\left\|s\left(t\right)\right\|+\overset{1}{\underset{i=0}{\sum }}\left[{\hat{K}}_{i}\left(t\right)-K_{i}^{*}\right]{\overset{˙}{\hat{K}}}_{i}\left(t\right)\\ =-{\left[\xi \left(t\right)-\xi \left(t-h\right)\right]}^{T}s\left(t\right)\\ -\overset{1}{\underset{i=0}{\sum }}\left[{\hat{K}}_{i}\left(t\right)-K_{i}^{*}\right]\left[{\left\|x\left(t\right)\right\|}^{i}\left\|s\left(t\right)\right\|-{\overset{˙}{\hat{K}}}_{i}\left(t\right)\right]. \end{array} \end{array}$$

Substituting (30) and (31) into (38), one can obtain

(39) $$\begin{array}{c} \overset{˙}{V}\left(E,t\right)\leq -{\left[\xi \left(t\right)-\xi \left(t-h\right)\right]}^{T}s\left(t\right)-\overset{1}{\underset{i=0}{\sum }}\left[{\hat{K}}_{i}\left(t\right)-K_{i}^{*}\right]\cdot \left[{\left\|x\left(t\right)\right\|}^{i}\left\|s\left(t\right)\right\|-\left\|s\left(t\right)\right\|\cdot {\left\|x\left(t\right)\right\|}^{i}+\alpha_{i}{\hat{K}}_{i}\left(t\right)\right]\\ =-{\left[\xi \left(t\right)-\xi \left(t-h\right)\right]}^{T}s\left(t\right)-\overset{1}{\underset{i=0}{\sum }}\left[{\hat{K}}_{i}\left(t\right)-K_{i}^{*}\right]\alpha_{i}{\hat{K}}_{i}\left(t\right)\\ =-{\left[\xi \left(t\right)-\xi \left(t-h\right)\right]}^{T}s\left(t\right)-\overset{1}{\underset{i=0}{\sum }}\alpha_{i}{\left[{\hat{K}}_{i}\left(t\right)-K_{i}^{*}\right]}^{2}+\overset{1}{\underset{i=0}{\sum }}\alpha_{i}{\left(K_{i}^{*}\right)}^{2}. \end{array}$$

Substituting (37) into (39), one can obtain

(40) $$\begin{array}{c} \overset{˙}{V}\left(E,t\right)\leq -\delta s^{T}\left(t\right)s\left(t\right)-\overset{1}{\underset{i=0}{\sum }}\alpha_{i}{\left[{\hat{K}}_{i}\left(t\right)-K_{i}^{*}\right]}^{2}+\overset{1}{\underset{i=0}{\sum }}\alpha_{i}{\left(K_{i}^{*}\right)}^{2}. \end{array}$$

Using the definition of *V*(*E*, *t*) in (33), condition (40) can be simplified to the following:

(41) $$\begin{array}{c} \overset{˙}{V}\left(E,t\right)\leq -\lambda V\left(E,t\right)+\overset{1}{\underset{i=0}{\sum }}\alpha_{i}{\left(K_{i}^{*}\right)}^{2}\\ =-\sigma V\left(E,t\right)-\left(\lambda -\sigma \right)V\left(E,t\right)+\overset{1}{\underset{i=0}{\sum }}\alpha_{i}{\left(K_{i}^{*}\right)}^{2}, \end{array}$$

where *λ*=2min{*δ*, *α*_0_, *α*_1_}, 0 < *σ* < *λ*.

It can be deduced that when *V*(*E*, *t*) ≥ (∑_*i*=0_^1^*α*_*i*_(*K*_*i*_^*∗*^)^2^/*λ* − *σ*), $\overset{˙}{V}\left(E,t\right)\leq -\sigma V\left(E,t\right)$.

*V*(*E*, *t*) will enter inside the ball in a finite time. From (41), it can be deduced that selecting small *α*_0_, *α*_1_ can reduce the size of the ball.

Therefore,

(42) $$\begin{array}{c} V\left(E,t\right)\leq \max\left\{V\left(0\right),\frac{\sum_{i=0}^{1}\alpha_{i}{\left(K_{i}^{*}\right)}^{2}}{\lambda -\sigma }\right\},\forall t\geq 0. \end{array}$$

Based on the Lyapunov stability theory, *V*(*E*, *t*) will enter inside the ball in finite time. The system is uniform ultimate bounded and independent of initial conditions.

A performance index is proposed to evaluate the parameters of the controller. It consists of the integration of the input and the error between the expected state and the output state. The integral interval should be large enough. The performance index *J* is defined as follows:

(43) $$\begin{array}{c} J=\frac{1}{t_{f}}\int_{0}^{t_{f}}{\left(x_{d}\left(t\right)-x\left(t\right)\right)}^{2}+\tau^{2}\left(t\right)\text{d}t. \end{array}$$

Adjust the appropriate control parameters to reduce the performance index as much as possible.

## 4. Simulation Studies

### 4.1. Example Introduction

In order to verify the control effect of the adaptive sliding mode control algorithm for two-wheel self-balancing vehicles with input delay, the main parameters of the two-wheel self-balancing vehicle used for testing are listed in Table 1.

### 4.2. Simulation Results

The experiments were carried out on Intel (*R*) Core (TM) i3-4150T CPU @ 3.00 GHz 3.00 GHz, a 64-bit operating system with 4.00 GB memory and an x64-based processor. The initial inclination angle of the system was 45^。^. The initial angular velocity was 0 degrees/s. The initial displacement was 0.2 m. The initial velocity was 0 m/s. The desired inclination angle was set to be 0 degrees. The desired angular velocity was 0 degrees/s. The desired displacement was 0 m. The desired velocity was 0 m/s.

**Figure 2:**
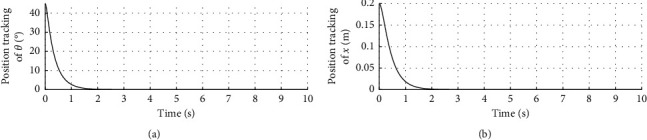
Response curve of inclination angle and vehicle position.

Figure 2 shows the response curve of inclination angle and vehicle position. The horizontal axis represents time in seconds. The longitudinal axis in the upper half of the figure represents the inclination angle of the vehicle in degrees. The longitudinal axis in the lower half of the figure represents the vehicle position in meters.

**Figure 3:**
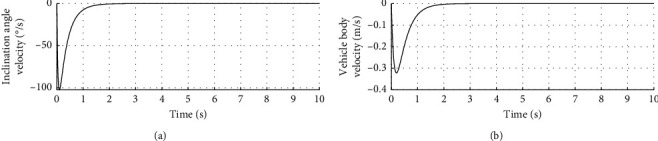
Inclination angle velocity and vehicle body velocity.

Figure 3 shows the inclination angle velocity and vehicle body velocity. The horizontal axis represents time in seconds. The longitudinal axis in the upper half of the figure represents the inclination angle velocity in °/s. The longitudinal axis in the lower half of the figure represents the vehicle body velocity in m/s.

**Figure 4:**
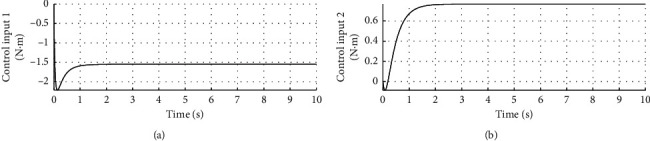
Control input curve.

Figure 4 shows the speed response curve of control input. The horizontal axis represents time in seconds, and the longitudinal axis represents the control input in N·m.

Figures 2–4 show that the adaptive sliding mode control can make the vehicle achieve autonomous balance.

### 4.3. Performance Comparison of Different Algorithms

In order to verify the effectiveness of the proposed algorithm, the control effects of the proposed algorithm are compared with other algorithms. The remaining parameters remain unchanged.

**Figure 5:**
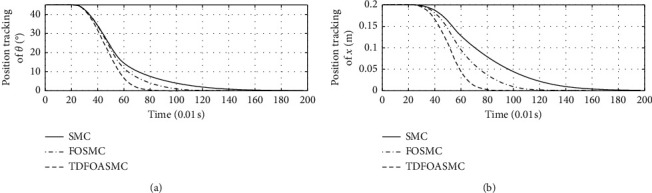
Inclination angle and vehicle position of different algorithms.

Figure 5 shows the response curve of inclination angle and vehicle position of different algorithms. The horizontal axis represents time in seconds. The longitudinal axis in the upper half of the figure represents the inclination angle of the vehicle in degrees. The longitudinal axis in the lower half of the figure represents the vehicle position in meters.

**Figure 6:**
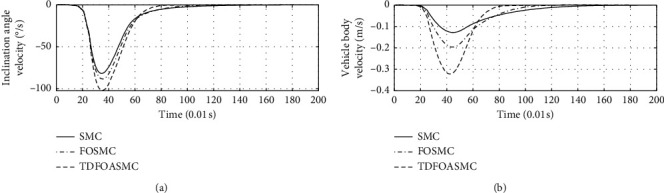
Inclination angle velocity and vehicle body velocity of different algorithms.

Figure 6 shows the inclination angle velocity and vehicle body velocity of different algorithms. The horizontal axis represents time in seconds. The longitudinal axis in the upper half of the figure represents the inclination angle velocity in °/s. The longitudinal axis in the lower half of the figure represents the vehicle body velocity in m/s.

**Figure 7:**
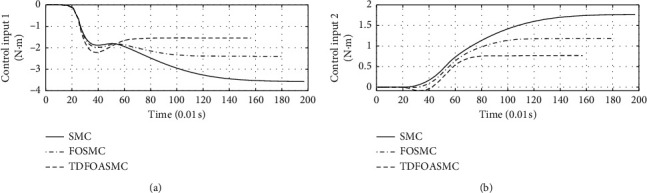
Control input of different algorithms.

Figure 7 shows the speed response curve of control input of different algorithms. The horizontal axis represents time in seconds, and the longitudinal axis represents the control input in N·m.

Figures 5–7 show that, compared with other algorithms, the proposed algorithm can achieve less adjustment time and require less control input.

### 4.4. Disturbance

**Figure 8:**
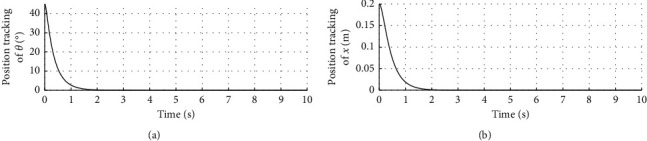
Inclination angle and vehicle position curve under disturbance.

In order to verify the effectiveness of the proposed algorithm under disturbance, set *d* = 0.2 × sin(3.5 × *t*). The remaining parameters remain unchanged.  Figure 8 shows the response curve of inclination angle and vehicle position under disturbance. The horizontal axis represents time in seconds. The longitudinal axis in the upper half of the figure represents the inclination angle of the vehicle in degrees. The longitudinal axis in the lower half of the figure represents the vehicle position in meters.

**Figure 9:**
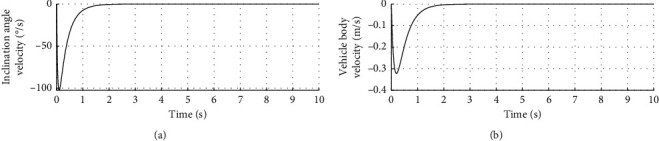
Velocity curve with disturbance.

Figure 9 shows the inclination angle velocity and vehicle body velocity under disturbance. The horizontal axis represents time in seconds. The longitudinal axis in the upper half of the figure represents the inclination angle velocity in °/s. The longitudinal axis in the lower half of the figure represents the vehicle body velocity in m/s.

**Figure 10:**
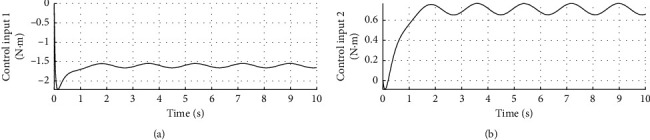
Control input curve with disturbance.

Figure 10 shows the speed response curve of control input under disturbance. The horizontal axis represents time in seconds, and the longitudinal axis represents the control input in N·m.

Figures 8–10 show when appropriate disturbance is introduced, the vehicle can adjust itself and quickly restore its stable state.

## 5. Experiment Results

**Figure 11:**
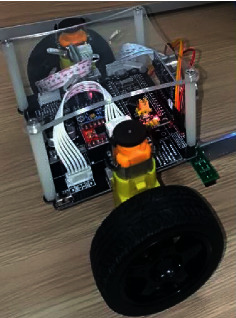
Two-wheel self-balancing vehicle system.

In this experiment, gyroscope MPU-6050 and accelerometer constituted a vehicle attitude detection device for a two-wheel self-balancing vehicle. A motor driving circuit was composed of a TB6612FNG chip. Fusion of the gyroscope data and accelerometer data was completed using a Kalman filter. The system used STM32F103x8B as the control core and completed processing of a sensor signal, realization of a filtering algorithm, body control, human-computer interaction, and so on. The vehicle could achieve autonomous balance without any intervention. When introducing appropriate disturbance, the vehicle could adjust itself and quickly restore its stable state. The vehicle could also complete basic movements, such as forward, backward, left turn, and right turn.  Figure 11 is a photo of the two-wheel self-balancing vehicle system.

The control circuit included ARM, three attitude sensors, filtering circuits, power circuit, power supply voltage conversion, and voltage stabilization. Its acceleration was measured by accelerometer, and the rotation angular velocity was measured by gyroscope. The motor used 7.4V DC.

**Figure 12:**
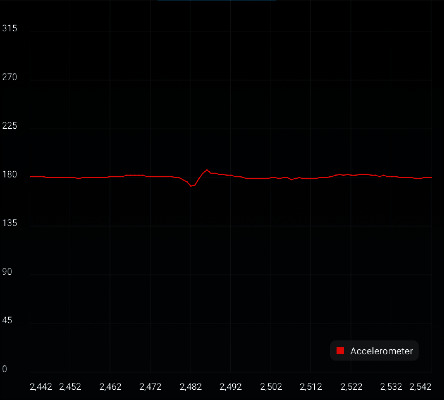
Acceleration curve.

Figure 12 shows the acceleration curve measured with an acceleration sensor. The horizontal axis represents time in seconds, and the longitudinal axis represents the acceleration in m/s^2^.

**Figure 13:**
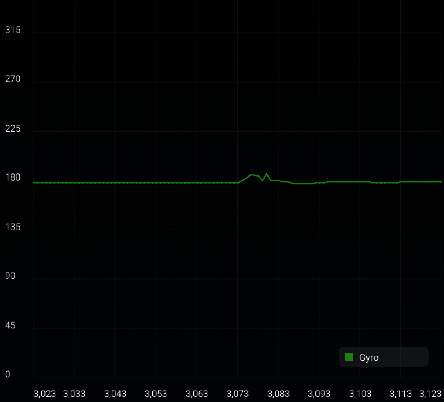
Angular velocity of rotation.

Figure 13 shows the angular velocity of rotation measured by a gyroscope. The horizontal axis represents time in seconds, and the longitudinal axis represents the angular velocity in ° /s.

**Figure 14:**
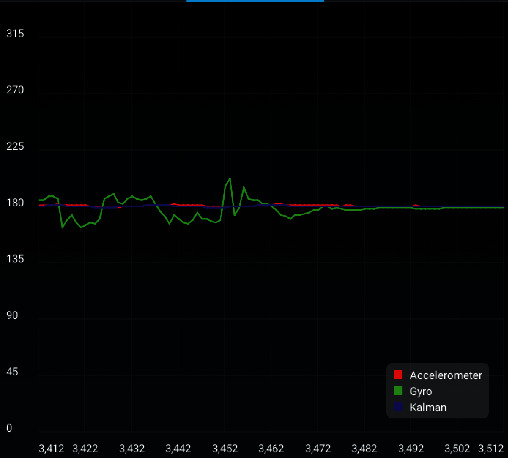
Acceleration, rotation angular velocity, and filtering curve.

Figure 14 shows acceleration, rotation angular velocity, and filtering curve. The horizontal axis represents time in seconds. The red line represents the acceleration in m/s^2^. The green line represents the angular velocity in °/s. The blue line represents the Klaman filtered acceleration in m/s^2^.

Figures 12to 14 show that the vehicle can adjust itself and restore its stability rapidly with the introduction of appropriate disturbance.

## 6. Conclusion

In this paper, a time-delayed fractional order adaptive sliding mode control algorithm for a two-wheel self-balancing vehicle system with input delay is proposed, and the stability of the closed-loop system is proved. The experimental results show that the adaptive sliding mode control algorithm can make the car achieve autonomous balance. When appropriate disturbance is introduced, the vehicle can adjust itself and quickly restore its stable state.

The next step in research should be to improve the control algorithm to better control accuracy and robustness of the two-wheel self-balancing vehicle.

## Figures and Tables

**Table 1:** Main parameters of two-wheel self-balancing vehicle.

Parameter	Value	Unit
*M*	9	kg
*m*	5	kg
*l*	0.95	m
*R*	0.2	m
*J*	12	kg·m^2^
*J*_*w*_	0.13	kg·m^2^
*τ*_1_	0.1	S

## Data Availability

All data, models, or codes generated or used during the study are available from the corresponding author by request. Most data and models generated or used during the study appear in the submitted article.
